# An Assessment of the Accuracy and Consistency of ChatGPT in the Management of Midshaft Clavicle Fractures

**DOI:** 10.7759/cureus.81906

**Published:** 2025-04-08

**Authors:** Christopha J Knee, Ryan J Campbell, Brahman S Sivakumar, Michael J Symes

**Affiliations:** 1 Department of Orthopaedics and Trauma Surgery, Royal North Shore Hospital, Sydney, AUS; 2 Department of Hand and Peripheral Nerve Surgery, Royal North Shore Hospital, Sydney, AUS

**Keywords:** artificial intelligence, chatgpt, clavicle operative fixation, midshaft clavicle fracture, patient education

## Abstract

Background

Midshaft clavicle fractures are common orthopaedic injuries with no consensus on optimal management. Large language models (LLMs) such as ChatGPT (OpenAI, San Francisco, USA) present a novel tool for patient education and clinical decision-making. This study aimed to evaluate the accuracy and consistency of ChatGPT’s responses to patient-focused and clinical decision-making questions regarding this injury.

Methods

ChatGPT-4o mini was prompted three times with 14 patient-focused and orthopaedic clinical decision-making questions. References were requested for each response. Response accuracy was graded as: (I) comprehensive; (II) correct but inadequate; (III) mixed with correct and incorrect information; or (IV) completely incorrect. Two consultant and two trainee orthopaedic surgeons evaluated the accuracy and consistency of responses. References provided by ChatGPT were evaluated for accuracy.

Results

All 42 responses were graded as (III), indicating a mix of correct and incorrect information, with 78.6% consistency across the responses. Of the 128 references provided, 0.8% were correct, 10.9% were incorrect, and 88.3% were fabricated. Only 3.1% of references accurately reflected the cited conclusions.

Conclusion

ChatGPT demonstrates limitations in accuracy and consistency when answering patient-focused queries or aiding in orthopaedic clinical decision-making for midshaft clavicle fractures. Caution is advised before integrating ChatGPT into clinical workflows for patients or orthopaedic clinicians.

## Introduction

Clavicle fractures are common, accounting for approximately 2-5% of all fractures in adults and 10-15% in children, with more than 80% involving the midshaft region [1,2]. These fractures predominantly occur in male patients and exhibit a bimodal distribution, with peaks among individuals under 25 years of age due to direct trauma and those aged over 55 years following falls [1,2]. Despite their prevalence, the optimal management of midshaft clavicle fractures remains a subject of conjecture. The primary treatment options include non-operative management, typically via a period of immobilisation in a sling, and operative intervention via open fracture reduction with internal fixation [1]. While the historical management of midshaft clavicle fractures has been primarily non-operative, recent studies indicate that operative fixation is associated with lower non-union rates and early functional recovery [3-6]. This evidence has been cited as contributing to a more than 300% increase in operative intervention rates over the past two decades in both male and female patients [7,8].

Traditionally, internet searches have served as a primary resource for patients seeking medical information. Amongst individuals attending orthopaedic outpatient clinics, approximately two-thirds report using the internet to obtain orthopaedic information, with over 80% using Google as their preferred search engine [9]. Recently, however, artificial intelligence (AI) chatbots - particularly large language models (LLMs) such as ChatGPT (OpenAI, San Francisco, USA) - have emerged as novel tools for accessing medical information. With over 200 million weekly active users, ChatGPT is one of the most widely used LLMs [10]. It is trained on a broad range of data, including publicly available content from the internet [11-13]. This training enables it to generate human-like responses across various topics, including healthcare. Accessible online, ChatGPT allows both patients and clinicians to engage in open-ended conversations and receive detailed responses to health-related questions [11-13].

The potential applications of ChatGPT within medicine and public health are extensive, encompassing personalised learning, medical and patient education, clinical decision-making, and documentation support [11]. Although ChatGPT demonstrates significant potential, its performance remains variable across different medical specialties. While it has shown greater proficiency in medical knowledge than other AI chatbots, it frequently generates inaccuracies, commonly referred to as “hallucinations,” which can mislead users [12,13]. These inaccuracies are particularly concerning as AI models become increasingly interactive and persuasive, rendering it difficult for users to discern the reliability of the information provided [13].

Therefore, the purpose of this study was to investigate the accuracy and consistency of ChatGPT's outputs for patient-focused inquiries and clinical decision-making related to midshaft clavicle fractures.

## Materials and methods

Study design

On August 31, 2024, ChatGPT-4o mini was prompted with a set of 14 questions addressing various aspects of midshaft clavicle fracture management (Table 1). Questions 1 to 4 were designed to replicate common patient searches related to clavicle fractures on Google. To achieve this, Google Trends (Google LLC, Mountain View, USA) - a tool used to identify the most popular search queries on specific topics - was utilised. The terms "broken collarbone", "broken clavicle", and "clavicle fracture" were searched to find the top related health queries worldwide from 2004 to the present. The most frequently associated search terms related to clavicle fractures, such as "sling", "displacement", "treatment", "surgery", and "recovery time", shaped the wording and focus of the patient questions. A 200-word limit was imposed to ensure responses remained concise and maintained reader engagement.

**Table 1:** ChatGPT input questions on the management of midshaft clavicle fractures

ChatGPT Input Questions	
Patient-Focused Questions	
1	In 200 words, outline the indications for surgical and non-surgical treatment of midshaft clavicle fractures, supported by evidence from four high-quality journal references. Ensure that the references are cited correctly both in-text and listed separately in a reference list.
2	In 200 words, compare the recovery times between surgical and non-surgical treatment of midshaft clavicle fractures, supported by evidence from four high-quality journal references. Ensure that the references are cited correctly both in-text and listed separately in a reference list.
3	In 200 words, compare the short- and long-term functional outcomes of surgical and non-surgical treatment of midshaft clavicle fractures, supported by evidence from four high-quality journal references. Ensure that the references are cited both in-text and listed separately in a reference list.
4	In 200 words, compare the benefits and risks of surgical versus non-surgical treatment of midshaft clavicle fractures, supported by evidence from four high-quality journal references. Ensure that the references are cited correctly both in-text and listed separately in a reference list.
Clinical Decision-Making Questions	
5	I’m an orthopaedic registrar seeking advice for a 10-year-old male with a midshaft clavicle fracture. His X-ray shows displacement of 100% of shaft width and 1 cm of shortening. Should I choose surgical or non-surgical treatment? Provide your 150-word recommendation with in-text citations and a separate reference list.
6	I'm an orthopaedic registrar seeking advice for a 23-year-old male with a comminuted midshaft clavicle fracture. His X-ray shows a Z-type fracture pattern. Should I choose surgical or non-surgical treatment? Provide your 150-word recommendation with in-text citations and a separate reference list.
7	I'm an orthopaedic registrar seeking advice for a 24-year-old male with a displaced midshaft clavicle fracture and significant skin tenting. Although he is open to surgery, he prefers non-surgical treatment if it is recommended. Should I opt for surgical or non-surgical treatment? Provide your 150-word recommendation with in-text citations and a separate reference list.
8	I’m an orthopaedic registrar seeking advice for a 35-year-old male with a midshaft clavicle fracture. His X-ray shows minimal displacement with a comminuted fragment. He is an avid cyclist and wants to return to full function quickly. Should I choose surgical or non-surgical treatment? Provide your 150-word recommendation with in-text citations and a separate reference list.
9	I’m an orthopaedic registrar seeking advice for a 35-year-old female with a midshaft clavicle fracture. She was initially treated with non-surgical management but returned to the clinic two weeks later with significant fracture displacement. Should I choose surgical or non-surgical treatment? Please provide your 150-word recommendation with in-text citations and a separate reference list.
10	I'm an orthopaedic registrar seeking advice for a 40-year-old female with a midshaft clavicle fracture. Her X-ray shows 3 cm of shortening and displacement greater than 100% of the shaft width. Should I choose surgical or non-surgical treatment? Provide your 150-word recommendation with in-text citations and a separate reference list.
11	I'm an orthopaedic registrar seeking advice for a 40-year-old male with a displaced midshaft clavicle fracture and a suspected subclavian vascular injury. He has poorly controlled diabetes and leads a sedentary lifestyle. Should I choose surgical or non-surgical treatment? Provide your 150-word recommendation with in-text citations and a separate reference list.
12	I'm an orthopaedic registrar seeking advice for a 60-year-old male with a displaced open midshaft clavicle fracture. He has pulmonary fibrosis, chronic kidney disease, and lives in a nursing home. Should I choose surgical or non-surgical treatment? Provide your 150-word recommendation with in-text citations and a separate reference list.
13	I'm an orthopaedic registrar seeking advice for a 60-year-old male with a displaced open midshaft clavicle fracture and a floating shoulder. He has pulmonary fibrosis, chronic kidney disease, and lives in a nursing home. Should I choose surgical or non-surgical treatment? Provide your 150-word recommendation with in-text citations and a separate reference list.
14	I’m an orthopaedic registrar seeking advice for a 70-year-old male with a midshaft clavicle fracture. His X-ray shows displacement of 100% of shaft width and 1 cm of shortening. He lives in a nursing home and has low functional demands. Should I choose surgical or non-surgical treatment? Provide your 150-word recommendation with in-text citations and a separate reference list.

Questions 5 to 14 were designed to simulate clinical decision-making scenarios that an orthopaedic clinician might present to ChatGPT for assistance with treatment decisions. The spectrum of management topics covered included clavicle fractures in paediatric and geriatric patients, comminuted fractures (both simple and complex), significant displacement and shortening, failed non-operative management, absolute surgical indications, and patient preferences. References were requested for all responses to assess the accuracy of the information and recommendations provided. For patient-focused questions, four references were requested to allow for sufficient analysis across responses.

Each question was submitted three times on the same day to assess response consistency. All entries were conducted in separate chat sessions to prevent prior interactions from influencing subsequent responses. Screenshots were taken to document the responses, with examples provided in Appendices (Figures 3-7).

Assessment of outputs

The responses were independently evaluated by two orthopaedic registrars (CK and RC) and two senior orthopaedic surgeons (MS and BS). An established accuracy grading scale was used to assess each response: (I) comprehensive; (II) correct but inadequate; (III) mixed with correct and incorrect information; and (IV) completely incorrect [14]. Each reviewer assessed consistency, defined by similar accuracy grading, content, and recommendations across the three responses for each of the 14 questions. Consistency was assigned a dichotomous grade of "yes" or "no," and any discrepancies in grading were resolved through discussion until a consensus was reached.

The accuracy of the references provided in the responses was verified through independent searches conducted in MEDLINE, Embase, and Scopus, including searches of article titles, abstracts, keywords, and authors. References were classified as incorrect if they could be located but contained inaccuracies (such as errors in the title, authors, publication date, or journal), or fabricated if they could not be located at all. Both correct and incorrect references were further analysed to determine whether the response accurately reflected the cited material.

Data analysis

Descriptive statistics, including frequencies, counts, percentages, and the standard error of the mean (SEM) for relevant variables, were calculated using Microsoft Excel v16.77 (Microsoft Corporation, Redmond, USA).

## Results

ChatGPT response characteristics

ChatGPT was presented with 14 questions, each repeated three times, generating 42 responses. The mean response length was 135.9 ± 3.9 words (SEM) (range: 108-197 words). A total of 128 references were provided with a mean of 3.0 references per response. ChatGPT consistently included in-text citations and reference lists as requested; however, in one response to Question 1, it provided only three references, despite being asked for four.

**Figure 1:**
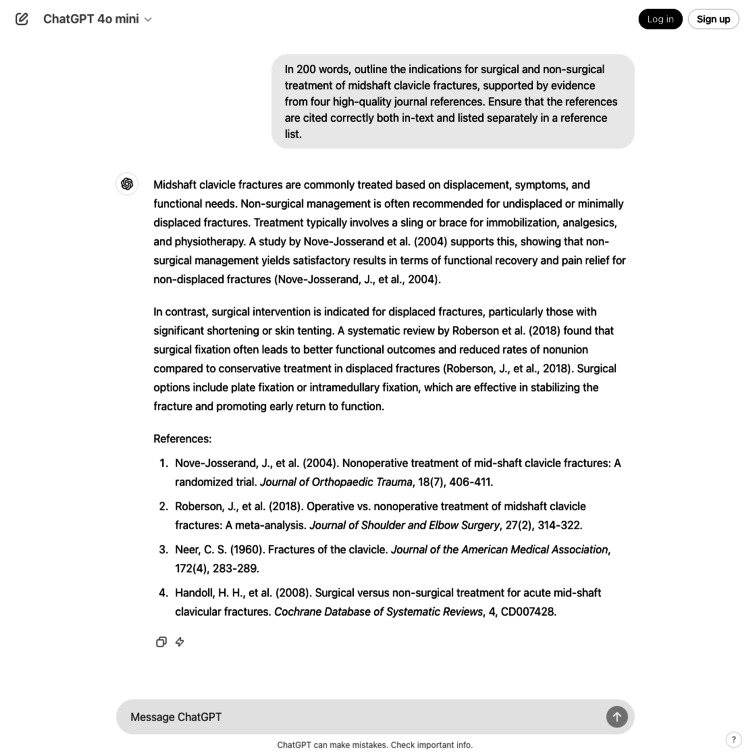
ChatGPT response to the indications for surgical and non-surgical treatment of midshaft clavicle fractures (Question 1, response 3)

**Figure 2:**
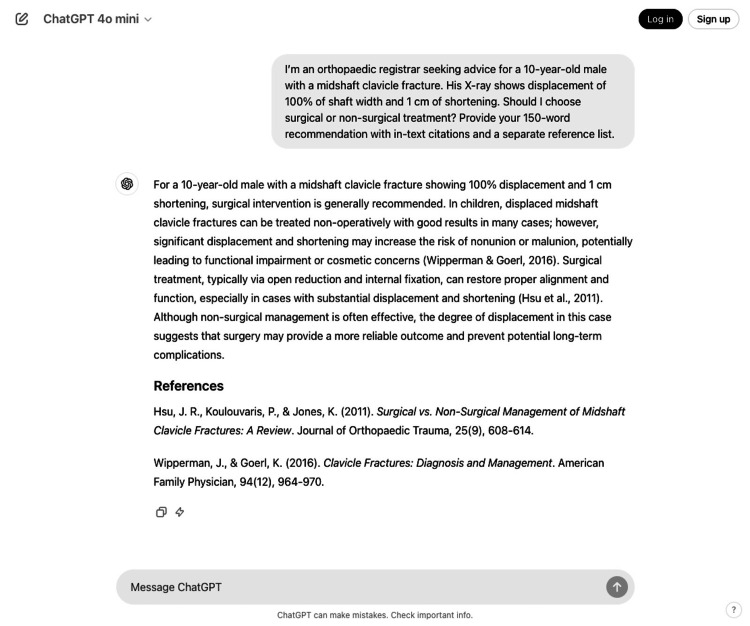
ChatGPT management recommendation for paediatric midshaft clavicle fracture (Question 5, response 2)

For patient-focused questions (Questions 1 to 4), the mean response length was 168.9 ± 6.6 words (SEM) (range: 123-197 words), with a mean of 3.9 references per response (range: 3-4). For clinical decision-making questions (Questions 5 to 14), responses averaged 122.7 ± 1.54 words (SEM) (range: 108-140 words), with 2.7 references per response (range: 2-4). Examples of ChatGPT responses to a patient-focused question and a clinical decision-making question are provided in Figures 1-2, respectively.

Information accuracy and consistency

The accuracy of all 42 responses was rated as Grade III, indicating a mix of correct and incorrect information. Consistency was observed in 11 of the 14 questions (78.6%) when comparing the three responses provided for each question (Table 2). There was agreement between graders regarding accuracy and consistency across all responses.

**Table 2:** Evaluation of the accuracy and consistency of ChatGPT's responses 'R1', first response; 'R2', second response; 'R3', third response; Ref, reference(s); 'n', number; 'nt', total number; 'Y', yes; 'N', no Grades I-IV: I. Comprehensive, II. Correct but inadequate, III. Mixed with correct and incorrect information, IV. Completely incorrect

Question	R1 Grade (I-IV)	R1 Ref (n/nt)	R2 Grade (I-IV)	R2 Ref (n/nt)	R3 Grade (I-IV)	R3 Ref (n/nt)	Consistency (Y/N)
1	III	0/4	III	0/3	III	0/4	Y
2	III	0/4	III	1/4	III	0/4	Y
3	III	0/4	III	0/4	III	0/4	Y
4	III	0/4	III	0/4	III	0/4	Y
5	III	0/2	III	0/2	III	0/3	N
6	III	0/3	III	0/3	III	0/3	Y
7	III	0/2	III	0/3	III	0/3	Y
8	III	0/3	III	0/3	III	0/2	N
9	III	0/3	III	0/3	III	0/2	Y
10	III	0/3	III	0/4	III	0/2	Y
11	III	0/3	III	0/3	III	0/3	Y
12	III	0/3	III	0/3	III	0/3	N
13	III	0/2	III	0/2	III	0/2	Y
14	III	0/2	III	0/4	III	0/2	Y

Reference analysis

Of the 128 references presented by ChatGPT, one was correct (0.8%), 14 contained incorrect details (10.9%), and 113 appeared fabricated (88.3%). Of the 15 references that could be verified (both correct and incorrect references), four (3.1%) accurately represented the research conclusions from the cited sources.

## Discussion

This study demonstrates the limitations of ChatGPT in providing accurate and consistent responses to both patient-focused inquiries and clinical recommendations for midshaft clavicle fractures. Notably, each response contained incorrect information, as well as fabricated references.

ChatGPT continues to see widespread adoption, with usage expanding rapidly across various sectors, including healthcare [10,12,13]. Its responses are becoming more sophisticated, making it increasingly difficult to distinguish AI-generated content from that of medical professionals [15]. This trend highlights the need for ongoing evaluation of ChatGPT's output accuracy and the credibility of its cited sources.

Kung et al. [16] reported that ChatGPT provided verifiable references 87.9% of the time when answering Orthopaedic In-Training Examination questions. In contrast, our study found that 88.3% of ChatGPT's references were fabricated, with only 11.7% being verifiable. A possible explanation for this discrepancy is that ChatGPT's reference accuracy may vary depending on the query type. Nonetheless, these findings emphasise the need for significant improvements in its ability to deliver consistent evidence-based information prior to incorporation in patient or clinician decision-making algorithms.

A recent systematic review and meta-analysis revealed that most evaluations of LLMs focus on medical knowledge tasks like answering licensing exam questions (44.5%) and making diagnoses (19.5%), while only 3.3% and 0.4% of these studies focused on orthopaedics and physical medicine, respectively. The authors recommended further studies that evaluate the use of clinical data, as well as expanding research into underrepresented specialties [17].

Evaluations of ChatGPT's performance in orthopaedics are mixed. Cuthbert and Simpson found that ChatGPT lacked accuracy and higher-order judgment when answering orthopaedic surgery and trauma-related questions, with 35.8% of answers being correct [18]. In contrast, Christy et al. [19] reported that 78% of ChatGPT’s responses to common distal radius fracture questions were deemed appropriate by a consensus of three fellowship-trained hand and upper-extremity surgeons. Truhn et al. [20] found that ChatGPT can provide largely accurate and clinically relevant treatment recommendations for common knee and shoulder conditions and is not prone to hallucinations. Seth et al. [21] assessed ChatGPT's effectiveness in generating information on hip osteoarthritis and reported that, although the responses were generally accurate, they were often superficial, lacked judgment, and omitted important research. Knee et al. [22] reported that early models of ChatGPT demonstrated significant limitations in accuracy and consistency when providing information on distal radius fractures. Other studies on carpal tunnel syndrome and scaphoid fractures have acknowledged ChatGPT's ability to provide validated information but highlighted inconsistencies in diagnostic pathways and clinical guidance [23,24].

In response to patient-focused questions in this study, ChatGPT generally recommended non-surgical management for non-displaced or minimally displaced midshaft clavicle fractures. Surgical management was consistently reported to offer benefits such as faster recovery, reduced non-union rates, and improved long-term functional outcomes. Whilst the literature supports these conclusions, the vast majority of the references provided by ChatGPT were fabricated. Additionally, ChatGPT failed to present a balanced discussion of evidence showing no significant long-term functional differences between surgical and non-surgical management [3,4,25]. Given that ChatGPT's knowledge extends to October 2023, it is unlikely that outdated input data accounts for this oversight [26]. Conflicting sources of information with varying weighting in ChatGPT's algorithms may be a contributory factor. Nevertheless, providing balanced information is crucial for facilitating collaborative, shared decision-making with patients, especially given the controversies surrounding the optimal management of clavicle fractures, and is a major limitation of the LLM tested.

The evaluation of ChatGPT's clinical decision-making on midshaft clavicle fractures revealed mixed results in terms of both accuracy and consistency. ChatGPT consistently recommended surgical management for Z-type fracture patterns, cases with skin tenting, failed non-surgical management, gross displacement, shortening, and subclavian vascular injury. It also recommended non-surgical management for geriatric patients with low functional demands. While these recommendations were generally appropriate, the reasoning provided often lacked depth and expert knowledge and frequently diverged from the evidence cited in its sources.

**Figure 3:**
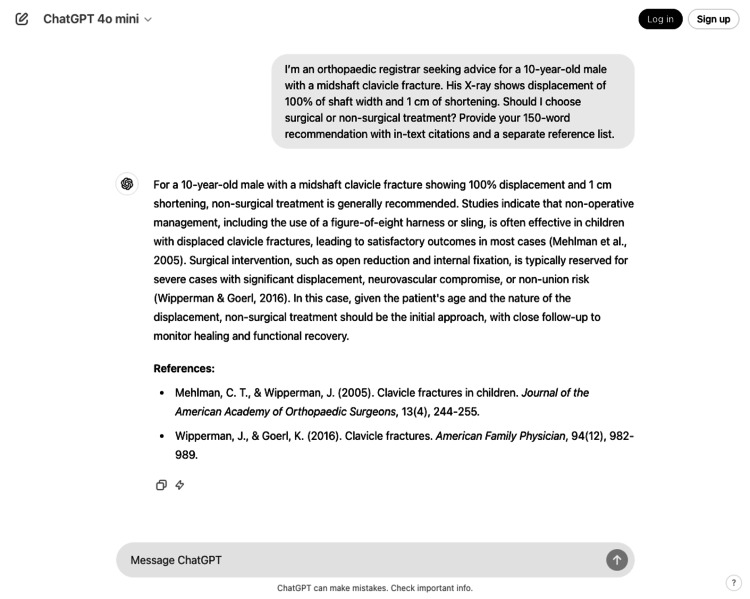
ChatGPT management recommendation for paediatric midshaft clavicle fracture (Question 5, response 1)

**Figure 4:**
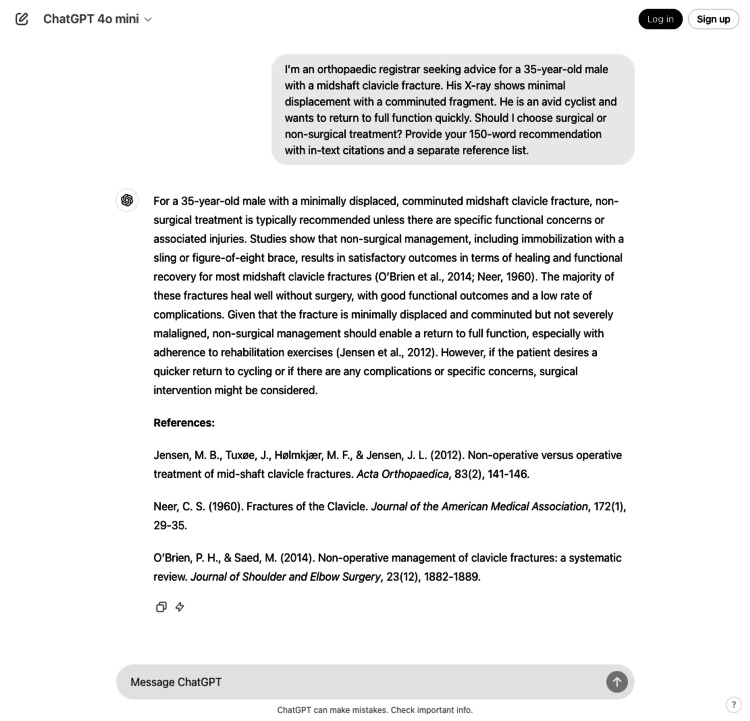
ChatGPT management recommendation for comminuted midshaft clavicle fracture (Question 8, response 1)

**Figure 5:**
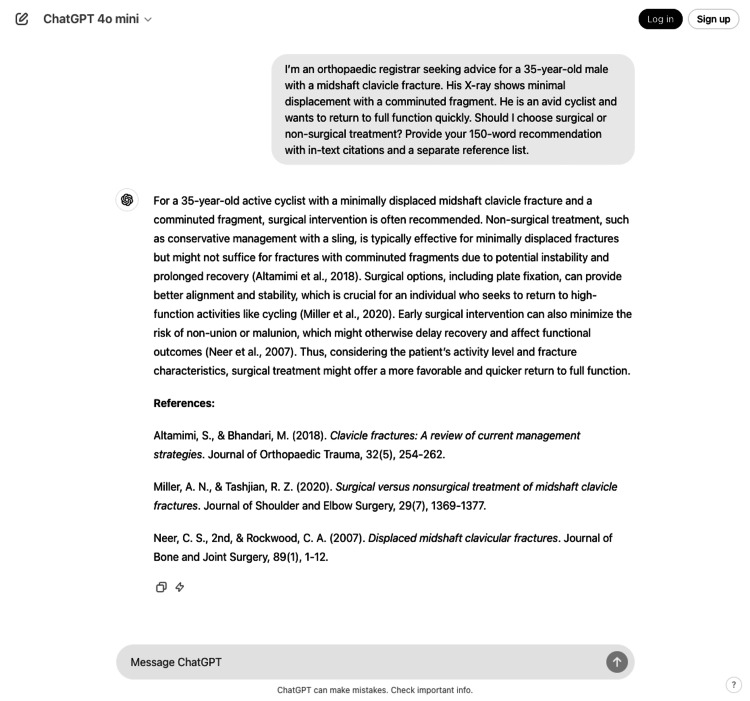
ChatGPT management recommendation for comminuted midshaft clavicle fracture (Question 8, response 2)

**Figure 6:**
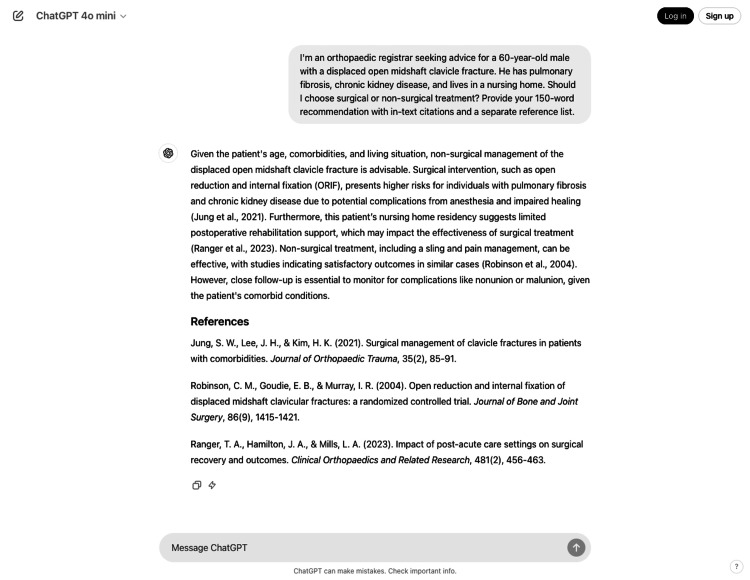
ChatGPT management recommendation for open midshaft clavicle fracture (Question 12, response 1)

**Figure 7:**
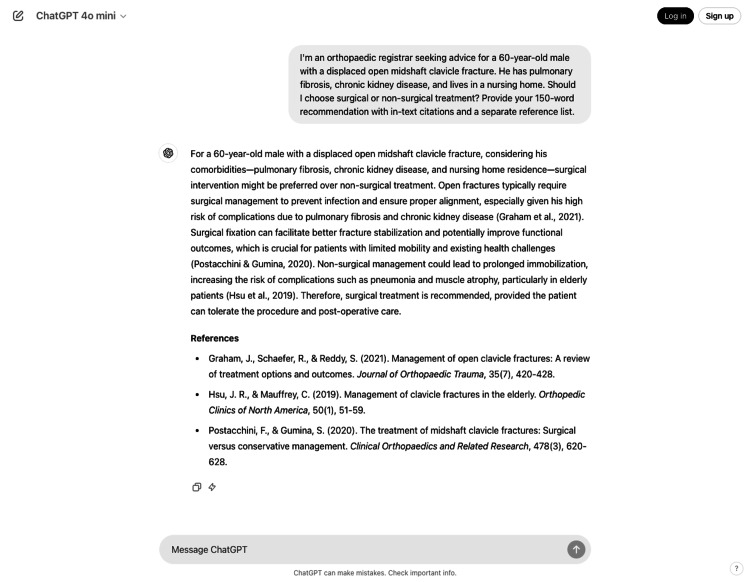
ChatGPT management recommendation for open midshaft clavicle fracture (Question 12, response 3)

ChatGPT did not consistently recognise open fractures as an absolute indication for surgery and provided inconsistent advice for paediatric patients and simple comminuted fractures (see Figures 3-7 within Appendices). It made decisions without clarifying important information about patient comorbidities. For instance, in both Questions 12 and 13 (involving open fractures in patients with chronic kidney disease and pulmonary fibrosis), ChatGPT failed to assess the severity of these conditions before recommending non-surgical management and did not suggest a multidisciplinary approach to preoperative planning. These findings align with those of Sparks et al. [27] who observed that ChatGPT often omitted critical information, contained inaccuracies, and lacked thoroughness in addressing treatment options and relevant risk factors in orthopaedic cases.

A strength of this study was the use of Google Trends to formulate patient-focused questions, incorporating the most commonly searched terms related to clavicle fractures on Google and enhancing relevance to typical patient queries. Including both patient- and clinician-focused questions enabled a comprehensive evaluation of ChatGPT’s utility across different user groups in orthopaedic care. Independent assessment by four orthopaedic reviewers reduced subjectivity in evaluating responses. Additionally, analysing the references provided by ChatGPT allowed for direct assessment of the accuracy of its responses and recommendations.

However, several limitations should be acknowledged. Although questions were based on commonly searched terms, they may not fully reflect how patients naturally phrase their queries when interacting with ChatGPT. Only single, isolated prompts were tested, which may not capture the more conversational or interactive nature of user engagement. Imposed word and reference limits may have restricted the depth of ChatGPT’s responses. Notably, when no reference limit was specified, ChatGPT often provided fewer citations. Finally, this study used ChatGPT-4o mini - the most accessible and widely available version - though results may vary with other versions of the model.

## Conclusions

This study found that ChatGPT has notable limitations in accuracy and consistency when providing patient information or recommending clinical decisions for midshaft clavicle fractures. Every response from ChatGPT contained incorrect or fabricated content. These findings highlight the need for patients and orthopaedic clinicians to exercise caution before integrating ChatGPT into clinical workflows.
